# Conjugate of Natural Bacteriochlorin with Doxorubicin for Combined Photodynamic and Chemotherapy

**DOI:** 10.3390/ijms25137210

**Published:** 2024-06-29

**Authors:** Ekaterina Plotnikova, Olga Abramova, Petr Ostroverkhov, Aleksandra Vinokurova, Dmitry Medvedev, Sergei Tihonov, Maksim Usachev, Anastasia Shelyagina, Anastasija Efremenko, Alexey Feofanov, Andrey Pankratov, Petr Shegay, Mikhail Grin, Andrey Kaprin

**Affiliations:** 1 National Medical Research Radiological Center of the Ministry of Health of the Russian Federation, 249036 Obninsk, Russia; olyabramova@gmail.com (O.A.); andreimnioi@yandex.ru (A.P.); dr.shegai@mail.ru (P.S.); kaprin@mail.ru (A.K.); 2 Department of Chemistry and Technology of Biologically Active Compounds, Medicinal and Organic Chemistry, Institute of Fine Chemical Technologies, MIREA-Russian Technological University, 119571 Moscow, Russia; mrp_ost@mail.ru (P.O.); dy.medvedev@mail.ru (D.M.); deviantprince13th@gmail.com (S.T.); maximus021989@mail.ru (M.U.); shelyna.lip@gmail.com (A.S.); michael_grin@mail.ru (M.G.); 3 State Budgetary Educational Institution School, 1329 Moscow, Russia; aleksvinokyrova@mail.ru; 4 Shemyakin-Ovchinnikov Institute of Bioorganic Chemistry of Russian Academy of Sciences, Miklukho-Maklaya str. 16/10, 117997 Moscow, Russia; aefr@mail.ru (A.E.);; 5 Faculty of Biology, Lomonosov Moscow State University, Leninskie Gori 1/12, 119234 Moscow, Russia

**Keywords:** photosensitizer, chemotherapy, photodynamic therapy, polytherapy, doxorubicin, natural bacteriochlorin

## Abstract

Chemotherapy is among the main classical approaches to the treatment of oncologic diseases. Its efficiency has been comprehensively proven by clinical examinations; however, the low selectivity of chemotherapeutic agents limits the possibilities of this method, making it necessary to search for new approaches to the therapy of oncologic diseases. Photodynamic therapy is the least invasive method and a very efficient alternative for the treatment of malignant tumors; however, its efficiency depends on the depth of light penetration into the tissue and on the degree of oxygenation of the treatment zone. In this work, a hitherto unknown conjugate of a natural bacteriochlorin derivative and doxorubicin was obtained. In vitro and in vivo studies showed a more pronounced activity of the conjugate against MCF-7 and 4T1 cells and its higher tumorotropicity in animal tumor-bearing animals compared to free anthracycline antibiotic. The suggested conjugate implements the advantages of photodynamic therapy and chemotherapy and has great potential in cancer treatment.

## 1. Introduction

Oncologic diseases constitute a group of drastic pathophysiological changes in the body that arise due to disruption of cell division. The mechanism of origination of malignant cells is quite complex and is related to many environmental factors (chemical and physical exposure, lifestyle, etc.); however, the key aspect of cancer occurrence is cell cycle disorders [1,2].

Photodynamic therapy (PDT) is the least-invasive method for cancer treatment, being highly efficient and widely used over the past decades [3,4]. Unfortunately, the use of PDT as a monotherapy has a number of limitations: inefficiency if the tumor focus is located deep due to the limited depth of light penetration into tissues; complexity of treatment of multiple tumors and disseminated cancer; and hypoxia of tumor tissue arising due to the specifics of tumor cell metabolism and oxygen consumption in the course of PDT [5].

Three main components are required for PDT: a photosensitive tumorotropic compound—the photosensitizer, a source of monochromatic radiation, and molecular oxygen. When a photoactive molecule is irradiated at the wavelength corresponding to the absorption maximum, generation of reactive oxygen species (ROS) occurs such as hydroxyl radicals, singlet oxygen, superoxide anions, etc. The elevated content of ROS in the cell leads to oxidative stress and initiation of apoptosis [6,7].

Chemotherapy is among the main treatment methods for patients with malignant neoplasms, both in the early stages of the disease and as palliative care [8,9,10,11,12,13,14]. Antitumor antibiotics are often used in treatment schedules for tumors of different nosology and localization. The most promising group is represented by anthracycline antibiotics; the most widely used agents of these include doxorubicin (DOX), daunorubicin [15], epirubicin [16], and mitoxantrone [17]. An enhanced sensitivity of tumor cells to these drugs is observed in the S-phase of the cell cycle since it is during this period that active DNA replication occurs. The mechanism of the cytotoxic effect has not been determined decisively. There are two points of view, according to which either antibiotic molecules generate semiquinone free radicals that are analogs of AOS in PDT, or anthracyclines intercalate between the DNA strands, thus disrupting the mitosis of tumor cells [18,19].

The use of chemotherapy or PDT as monotherapies is inefficient so combined methods of oncology treatment are nowadays used in clinical practice [20,21].

Combining PDT with radiation therapy increases the efficiency of tumor irradiation due to a reduction in tumor size and weakening of the antioxidant protection of tumor cells upon irradiation [22]. Intraoperative PDT reduces the invasiveness of surgical intervention and expands the options for the treatment of tumors with difficult anatomical localizations, including tumors of the brain, cervical spine, and organs with complex vasculature [23]; moreover, it improves the visualization of the tumor node, which allows affected tissues to be removed with the smallest damage to the healthy ones [24]. Numerous studies have confirmed the efficiency of combining PDT with other methods of cancer treatment. Apparently, the positive effect is related to its impact on various biochemical pathways of cancer cell life and division that cannot be achieved using monotherapy [25].

Combining PDT with chemotherapy is a very promising approach since chemotherapeutic agents demonstrate low selectivity. The tumorotropicity of modern photosensitizers and the damage to tumor cells by the ROS increase the sensitivity of affected tissues to chemotherapeutic drugs, and hence, make it possible to reduce the therapeutic dose of the drug and achieve a better therapeutic result [26]. Moreover, the possibility of overcoming multidrug resistance, which greatly limits the use of chemotherapy, was reported [3].

In this work, DOX was selected as an efficient broad-spectrum antitumor antibiotic [27,28,29,30,31]. It is produced by the Streptomyces coeruleorubidus or Streptomyces peucetius microorganisms or obtained semi-synthetically from daunorubicin [32]. Despite its high efficiency, DOX, like all anthracycline antibiotics, has the very serious drawback of low selectivity and specificity for a tumor, which results in the development of serious adverse reactions including damage to many organs and tissues. Cardiotoxicity is a limiting factor that prevents the escalation of a cytostatic dose, and hence, attainment of high efficiency [33]. To overcome the considered limitations, a conjugate of bacteriochlorin and DOX was prepared (PS+DOX). This PS absorbs in the near-infrared region of the spectrum where the penetration of light into tissues is the deepest, which allows deep-seated invaded tissues to be treated [34,35,36].

The main drawback of chemotherapy is the lack of targeted delivery of chemotherapeutic agents to the tumor, which leads to high systemic toxicity and numerous side effects. Binding an anthracycline antibiotic to bacteriochlorin that has high tumorotropicity—as shown in many PDT studies—allows the targeted delivery of the antibiotic to a tumor, thereby reducing its toxic effect on the body by reducing the therapeutic dose.

On the other hand, the low oxygenation of tumor tissues, including zones of hypoxia in the central regions of solid tumors, is a limitation of PDT.

The action of chemotherapeutic drugs, including anthracycline antibiotics, is not related to the presence or absence of oxygen in the tissue. Since for most of them, the main target is the genetic apparatus of the cell, the cytotoxic effect is realized mainly in relation to cells with increased proliferation.

To estimate the efficiency of antitumor therapy with the conjugate (PS+DOX) obtained, its combined photodynamic and chemotherapeutic effects on MCF-7 and 4T1 cells and sarcoma M-1 tumor were studied and compared to those of compound **1** (PS) and DOX in monotherapy using each drug alone.

## 2. Results and Discussion

### 2.1. Synthesis and Physicochemical Properties of Compound **2** (PS+DOX)

**Scheme 1:**
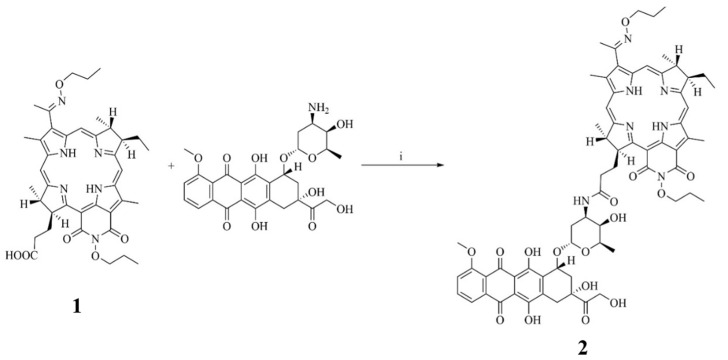
Preparation of the conjugate of bacteriochlorin derivative and Doxorubicin: (i) HBTU, Et_3_N, DMF, 12 h.

Addition of Doxorubicin to bacteriopurpurinimide was performed by creating an amide bond between the carboxyl group of PS and the amino group of Doxorubicin using the activated ester method. Due to the spatial hindrance of the carboxyl and amino groups, a high yield of the target product was achieved only with HBTU, while the other known reagents (carbodiimides) were inefficient (Scheme 1).

**Figure 1:**
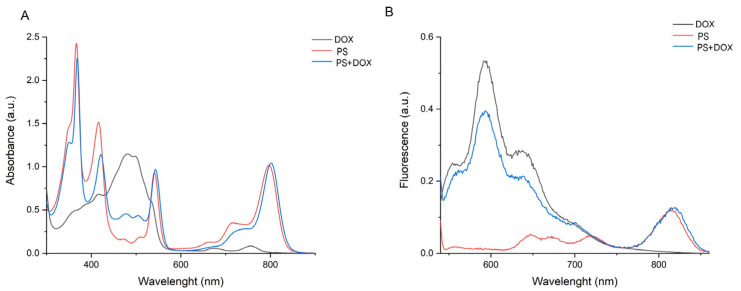
Absorption ((**A**,**B**): PS+DOX—41.5 μM; PS—31.3 μM; DOX—121.1 μM) and fluorescence (**B**) spectra of DOX, PS, and PS+DOX.

The spectra of PS, PS+DOX, and DOX were recorded. The spectrum of PS+DOX contains a band characteristic of Doxorubicin in the region of 450–520 nm, whereas the spectrum of PS demonstrates no absorption in this region (Figure 1).

A combination of physicochemical methods of analysis, including high-resolution chromatography–mass spectrometry and NMR spectroscopy, was used to confirm the structure of PS+DOX reliably.

**Figure 2:**
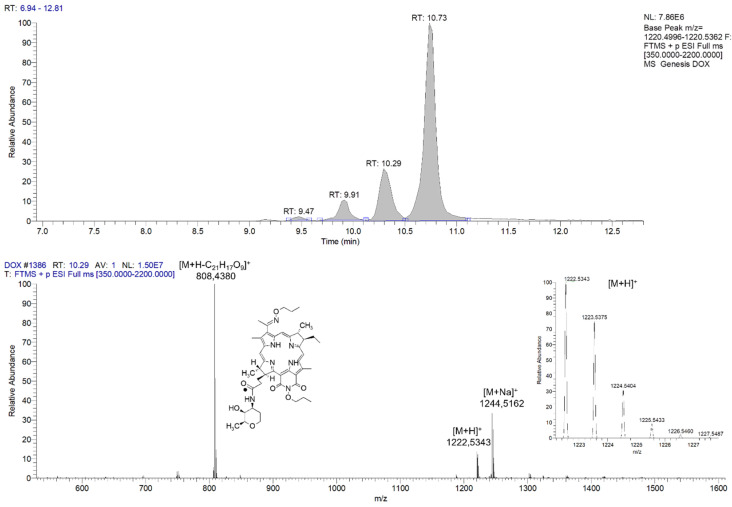
Mass chromatogram and first-order mass spectrum of PS+DOX.

Figure 2 shows the mass chromatogram and first-order mass spectrum of PS+DOX. The calculated *m*/*z* value of C_66_H_77_N_7_O_18_ [M+H]^+^, which is 1223.54, matches the value of 1223.5375 [M+H]^+^ obtained.

The mass spectrum shows signals of the molecular ion, a sodium PS+DOX adduct, and fragments formed upon breaking the O-glycoside bond. The presence of several peaks with different retention times on the chromatogram is explained by the presence of optical isomers at different positions since the monoisotopic composition and spectral characteristics of the fractions did not differ.

### 2.2. In Silico Study of the Interaction of PS+DOX with Biological Targets

Previously, our research team found correlations between the docking of platinum-containing agents into a DNA molecule and the results of in vitro biological studies. In this work, we studied the interaction of the compounds obtained with DNA since this is the main molecular target for Doxorubicin [37,38]. To predict the mechanism of interaction between the target compound and DNA, we docked it into the DNA sequence d(CAAAAATTTCTTCTGAGTTTTTTTTTTTTT)_2_ obtained from the 1E9J complex. Topoisomerase II protein, which is also a target for doxorubicin and its analogs, was considered an additional target [39]. The 1ZXM model was used.

The 3D structure of Doxorubicin was taken from the drugbank.com database (DB00997).

**Figure 3:**
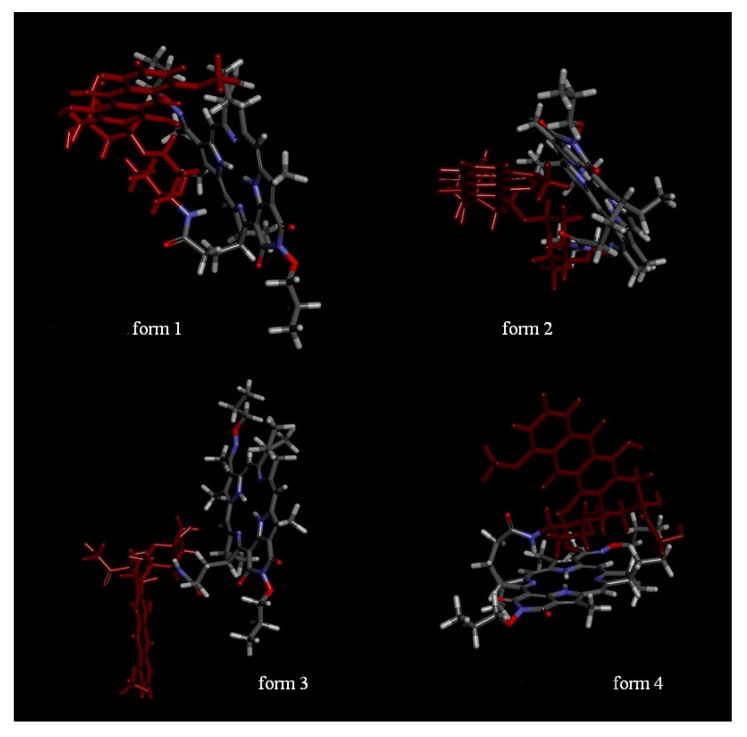
Assumed structures of the conjugates; **form 1, form 2**—intramolecular interactions between the doxorubicin and PS moieties exist; **form 3**—DOX and PS do not interact; **form 4**—there are intramolecular interactions between the PS and the glycoside moiety.

Optimization of the 3D structure of the PS+DOX yielded four stable structures with E = 142.8715 ± 16.7478 (Figure 3), which differed in the position of the Doxorubicin moiety with respect to the macrocycle.

**Figure 4:**
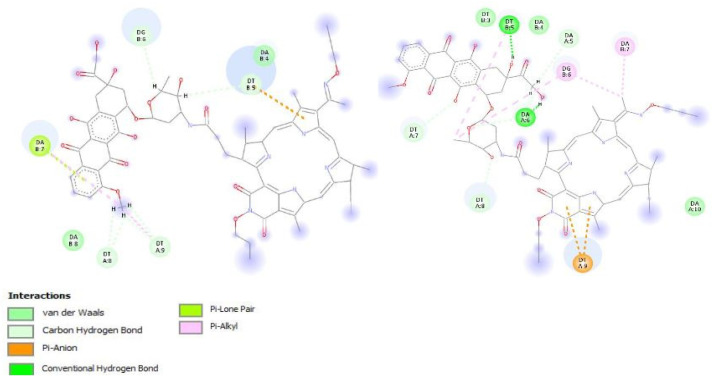
Two-dimensional diagrams of the interaction of PS+DOX and a DNA fragment.

All the structures obtained were used in the docking. Special attention was paid to the ligand poses, in which the Doxorubicin moiety was located in DNA grooves and had a larger number of interactions than the macrocyclic moiety. The mean E_total_ and E_shape_ energies for suitable poses of the structures obtained were used to estimate the conjugate–DNA interactions. An insignificant increase in the total binding energy and surface interaction energy were observed. Moreover, an increase in the number of hydrogen bonds to 8 was observed. In addition, the π–anionic interaction of exocycle E and pyrrole C with the oxygen atom of the phosphate group, which may increase the stability of the conjugate-DNA complex, was observed for all the structures (Table 1, Figure 4).

Binding into the minor groove of the DNA fragment—which is characteristic of such conjugates—was mostly observed.

The study of the interaction of the conjugates with topoisomerase II by the “blind” docking method showed no significant differences but the total energy was much higher for PS+DOX at −320.50 kJ/mol and −435.50 kJ/mol for DOX and PS+DOX, respectively.

**Figure 5:**
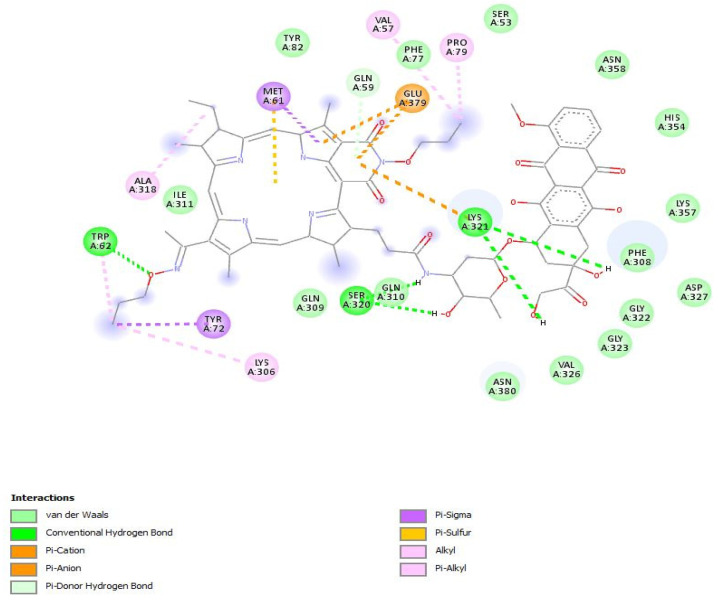
Two-dimensional diagram of the interaction of the PS+DOX and the active site of top2a.

To study the interactions in more detail, docking into the active site of the protein was performed. The affinity of the conjugate to the protein was similar to that of Doxorubicin X in terms of energy and amounted to −10.5 kcal/mol and −10.0 kcal/mol, respectively. However, their positions in the active site differed: the conjugate was turned by the macrocycle moiety to the active site and interacted with the same amino acids as Doxorubicin (Figure 5). In the case of docking into the two subunits of the top2a protein, the affinity of the conjugate to the protein in terms of energy was higher than that of Doxorubicin and amounted to −9.7 kcal/mol and −8.5 kcal/mol, respectively.

Of all the structures, form 4 of PS+DOX was the most probable one for docking into the active site. Based on the data obtained, it can be predicted that the conjugate obtained would exhibit the properties of a CT agent whose activity would not be lower than that of the initial doxorubicin.

### 2.3. Determination of the Quantum Yield of Singlet Oxygen Generation by PS+DOX in Solution

**Figure 6:**
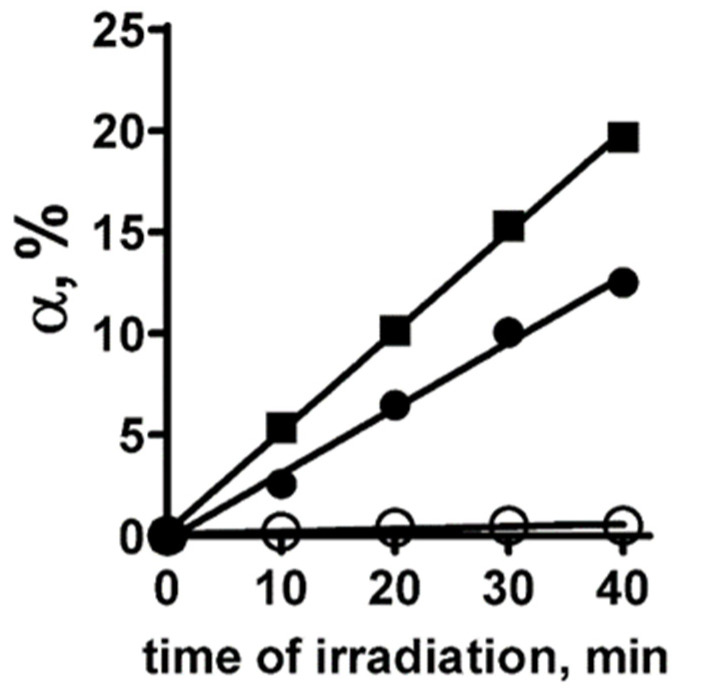
Photoinduced production of singlet oxygen by PS+DOX in 1% CrEL that was measured using the method of chemical traps. Graphs show the time course of bleaching of RNO molecules (α, the percentage of bleached RNO molecules) induced by the reaction of singlet oxygen with histidine in the presence of Rose Bengal (standard having the quantum yield of singlet oxygen production of 0.75) (■), PS+DOX (●), PS and sodium azide (○) during photoirradiation of solution.

To study the photoinduced generation of singlet oxygen by PS+DOX, the method of chemical traps was used [40]. The quantum yield of singlet oxygen (*φ*) from PS+DOX was determined in a solution using the 4-nitroso-N,N-dimethylaniline-histidine assay and Rose Bengal (RB) as the standard, which has *φ* = 0.75 [41,42]. In this assay, the interaction of singlet oxygen with histidine leads to the formation of a peroxide intermediate, which, in turn, induces bleaching of 4-nitroso-N,N-dimethylaniline (RNO). The measurements were performed for PS+DOX dissolved in a 1% emulsion of Cremophor EL (CrEL), which stabilized the monomeric form of PS+DOX in aqueous solution. Indeed, irradiation of PS+DOX results in the generation of singlet oxygen (Figure 6). In confirmation of this, the photoinduced reaction of RNO decoloration in the presence of histidine is totally suppressed by sodium azide, a well-known singlet oxygen quencher (Figure 6). The *φ* of PS+DOX is 0.45 ± 0.03.

### 2.4. Cellular Imaging of PS+DOX In Vitro

**Figure 7:**
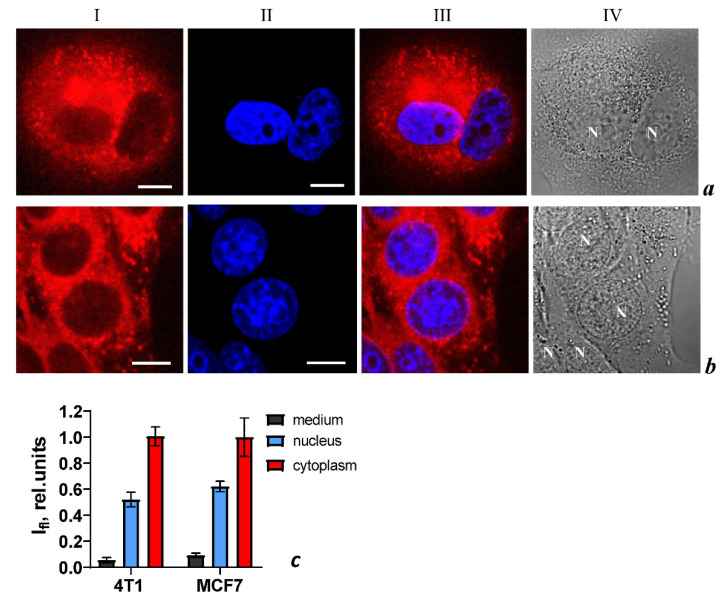
Intracellular distribution of PS+DOX (column **I**) and DNA probe Hoechst 33342 (column **II**) in MCF-7 (row **a**) and 4T1 (row **b**) cells measured by laser scanning confocal microscopy. Cells were incubated with PS+DOX (5 μM) for 4 h followed by incubation with Hoechst 33342 (3 μM) for 1 h. The bar represents 10 μm. (column **III**) Merged images of PS+DOX and Hoechst 33342. (column **IV**) Transmitted light images of cells. (**c**) Average intensities of fluorescence of PS+DOX in the medium (background outside the cells), in nuclei, and in cytoplasm of 4T1 and MCF-7 cells. The data are presented as mean ± SD (*n* = 30 ± 5 cells in each sample group).

Using confocal laser scanning microscopy (CLSM), it was found that PS+DOX penetrated into MCF-7 and 4T1 cells. Similar intracellular distributions of the compound are observed in both types of cells: accumulation in vesicular structures, intense diffuse staining of the cytoplasm, and significantly weaker staining of the nucleus (Figure 7). The intracellular distributions of the PS+DOX are similar after 4 and 24 h incubation of cells with PS+DOX.

**Figure 8:**
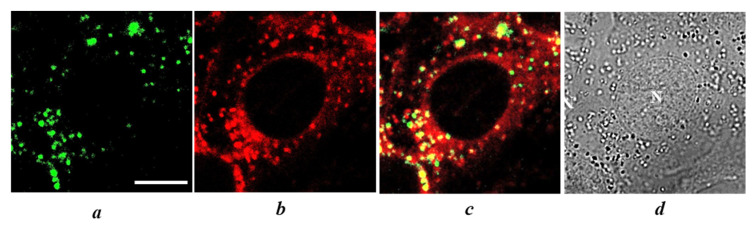
Staining of lysosomes with LG in living 4T1 cells loaded with PS+DOX. (**a**,**b**) Intracellular distributions of LG (**a**) or PS+DOX (**b**) recorded using CLSM. (**c**) Overlap of (**a**,**b**) showing the intracellular distribution of LG (green) and PS+DOX (red). Yellow color indicates colocalization of PS+DOX and LG in lysosomes. (**d**) Transmitted light image of cells. The bar represents 10 μm. N marks a nucleus.

To clarify the origin of vesicles accumulating PS+DOX, the cells were co-incubated with PS+DOX and LysoTracker Green (LG), a vital fluorescent probe of lysosomes. Colocalization analysis revealed the accumulation of PS+DOX in lysosomes of MCF-7 and 4T1 cells (Figure 8). The colocalization coefficients for PS+DOX and LG (i.e., lysosomes), which were calculated using the colocalization threshold plugin of the ImageJ program (National Institute of Health, Bethesda, MD, USA), were 0.64 ± 0.06 and 0.68 ± 0.4 for MCF-7 and 4T1 cells, respectively.

### 2.5. Photo- and Cytotoxicity In Vitro

To estimate photoinduced activity, PS+DOX was incubated with MCF-7 and 4T1 cells for 4 h (optimal time for accumulation in tumor cells), followed by irradiation. Survival was assessed 24 h later. To evaluate cytotoxic activity, PS+DOX and DOX were incubated for 72 h with cells without irradiation. To estimate the combined effect (photoinduced and cytotoxic), cells were incubated for 4 h with the PS, irradiated under the same conditions, and then incubated for 72 h to realize the cytotoxic effect. The results of these studies are presented in Table 2.

A study of the photoinduced activity of PS+DOX revealed the inhibition of proliferation of human mammary adenocarcinoma MCF-7 cells, IC50 was 671 ± 14 nM, and of mouse mammary carcinoma 4T1, where IC50 was 188 ± 8 nM. Increasing the incubation time after irradiation to 72 h resulted in a 1.5-fold decrease in IC50 both for MCF-7 and 4T1, which amounted to 442 ± 11 nM and 123 ± 7 nM, respectively. This indicated the realization of the additive effect due to both the bacteriochlorin molecule and Doxorubicin (Table 3). Statistically significant differences are observed between the photoinduced activity and combined activity groups for MCF-7 and 4T1 at *p*-value < 0.0001 according to the ANOVA analysis followed by Bartlett’s test.

When irradiating PS, 4 h after exposure to wells with MCF-7 and 4T1 cells, photoinduced activity was detected—the IC50 was 707 ± 16 nM and 194 ± 10 nM; therefore, increasing the incubation time after irradiation to 72 h does not lead to a significant IC50 measurement (*p* > 0.05) (Table 2).

The incubation of DOX with MCF-7 and 4T1 cells for 72 h revealed an cytotoxic effect, the IC50 was 5525 ± 71 and 6237 ± 96 nM, respectively.

It should be noted that 4T1 mouse mammary carcinoma cells were more sensitive to the action of PS, PS+DOX, and DOX.

When comparing the combined activity of PS and PS+DOX, a higher activity of PS+DOX was revealed, the IC50 for MCF-7 cells is 1.6 times higher, for 4T1—1.5 times.

Thus, high activity against breast tumor cells was demonstrated for PS+DOX owing to the combined effect of photodynamic therapy and chemotherapy.

### 2.6. Study of the Dynamics of PS, PS+DOX, and DOX Accumulation in a Tumor and in Healthy Tissue

Investigating the accumulation of various photoactive substances in cells is an important aspect of screening studies that makes it possible to optimize the conditions of photodynamic treatment. Determination of the drug–light interval (DLI), i.e., the time from the moment of PS administration to that of laser irradiation, allows the treatment at a high level of PS accumulation in the tumor (sarcoma M-1) while minimizing its concentration in normal tissues. The dynamics of drug accumulation in the M-1 tumor and in the surrounding tissues upon intravenous administration at a dose of 5.0 mg/kg was studied by local fluorescence spectroscopy for DOX, PS, and PS+DOX at different wavelengths.

For all the compounds, an increase in their levels in both tumor and surrounding tissue is observed 30 min after the intravenous administration.

**Figure 9:**
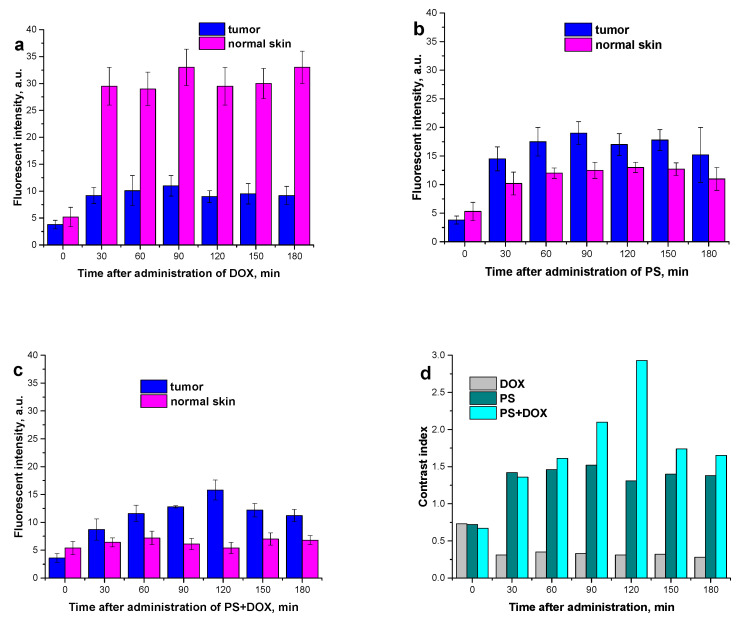
Dynamics of drug accumulation in the M-1 tumor and in surrounding thigh tissues upon intravenous administration at a dose of 5.0 mg/kg: (**a**) DOX; (**b**) PS; (**c**) PS+DOX; (**d**) contrast index (fluorescence intensity tumor/healthy tissue).

For DOX, the greater increase in drug accumulation in the healthy tissue is statistically significant not only compared to the baseline values (intrinsic fluorescence of biological tissues (*p* < 0.001)) but also compared to the tumor tissue (*p* < 0.001) (Figure 9a).

For PS, the increased accumulation by the tumor tissue compared to the surrounding healthy tissue (*p* < 0.05) begins as early as in 30 min. The maximum contrast index is 1.5. The optimal DLI is from 30 min to 180 min (Figure 9b).

PS+DOX shows the maximum accumulation in the tumor after 120 min with a contrast index of 3. PS+DOX appears to be preferentially accumulated by the tumor. This indicates that the biodistribution of DOX is more selective when it is conjugated with the photosensitizer. Thus, the optimal DLI is between 90 min and 120 min, with the smallest accumulation of the substance in the surrounding healthy tissue within this period (Figure 9c).

### 2.7. Specific Activity In Vivo

Since the studied PS and PS+ DOX has an absorption in the region of 800 nm—which allows it to affect larger and deeper-lying tumors—in vivo studies were conducted on rats with an M-1 tumor.

**Figure 10:**
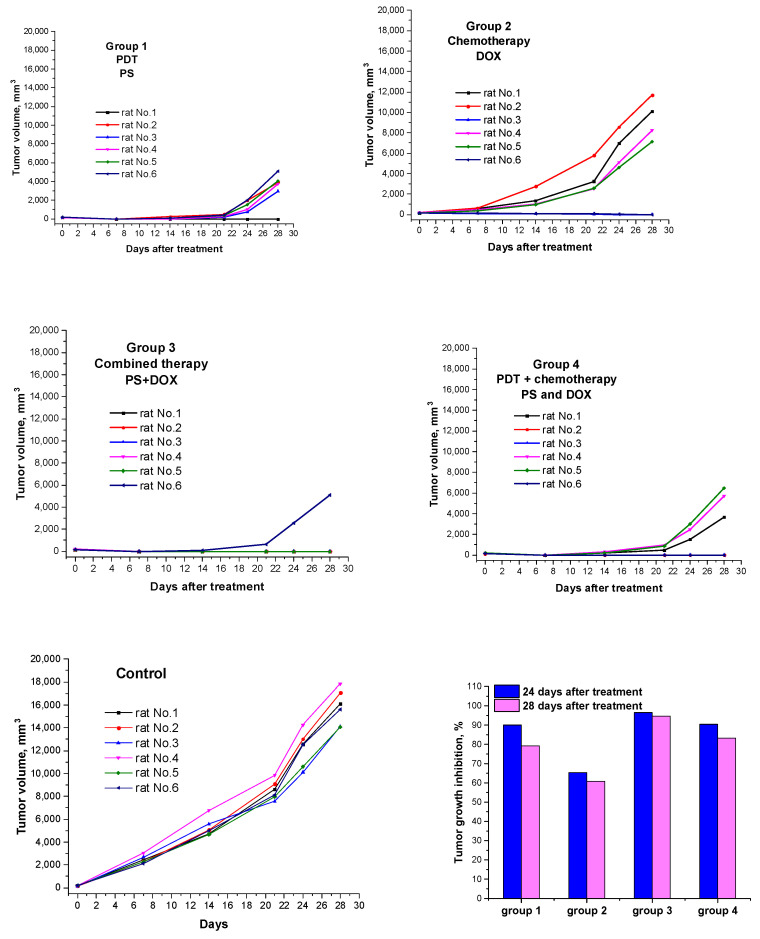
Tumor volume growth curves for individual rats in each treatment group (*n* = 6). Tumor volume was estimated as V = d_1_ × d_2_ × d_3_ × 0.52, where d_1_, d_2_, and d_3_ are three orthogonal diameters of the tumor nodule. Tumor growth inhibition (TGI) values calculated by the formula [(Vc − Vex)/Vc] × 100%, where Vex and Vc are the average tumor volumes in the experimental and control groups (*n* = 6).

**Figure 11:**
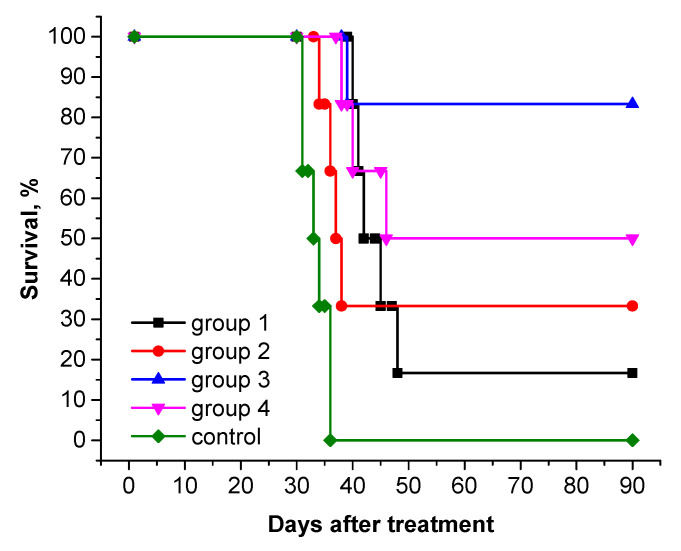
Kaplan–Meier survival curves in each treatment group (*n* = 6).

To estimate the antitumor efficacy of PS+DOX, we conducted a pilot study in an in vivo system and studied its combined photodynamic and chemotherapeutic properties in the treatment of rats with subcutaneous tumor M-1 sarcoma. The results obtained are presented in Table 3 and Figure 10. The survival rate of animals after therapy is shown in Figure 11 (Kaplan–Meier).

After the PDT session, limb swelling was observed in the animals, which persisted until 5–6 days after exposure. Animal death was not observed in any of the experimental groups.

Treatment schedule I

PDT with PS (dose 2.76 mg/kg with laser exposure parameters E = 150 J/cm^2^; Ps = 0.48 W/cm^2^) to a pronounced antitumor effect: the tumor growth inhibition (TGI) in 24 days after treatment reached 90.0%, while it decreased to 79.2% in 28 days; the life expectancy of rats was higher than in the control group (Table 3, Figure 10); complete regression of the tumor focus was observed in one animal (rat No. 1) (RN 16.7%).

Treatment schedule II

After a course of chemotherapy with DOX at a dose of 2.24 mg/kg, a slow increase in tumor volume was observed in rats with M-1 sarcoma compared with tumors in the control group; the TGI on days 24 and 28 after treatment was 65.4 and 60.8%, respectively. On day 90 after therapy, complete regression of the tumor focus was observed in 33.3% of animals (rats No. 1 and 6) (Table 3, Figure 10).

Treatment schedule III

After combination therapy with PS+DOX at a dose of 5.0 mg/kg, a high antitumor effect was observed: TGI on the 28 th day after treatment was 94.6%; the average life expectancy (MLE) of animals was 81.3 ± 17.0 days; the percentage of cured animals on day 90 after therapy was 83.3%; continued tumor growth was observed in only one rat (No. 6); the increase in life expectancy (LEI) reached 150.3%.

Treatment schedule IV

Combination therapy with simultaneous administration of DOX and a PDT session with PS led to a less-pronounced antitumor effect than in group 3: the LEI was 100.5% and the number of cured animals was 50% (rats No. 2, 3, and 6).

Thus, the results obtained indicate an increase in antitumor efficacy in rats with M-1 sarcoma after combination therapy with PS+DOX: MLE—81.3 ± 17.0 days; LEI—150.3%; and RN—83.3%.

## 3. Materials and Methods

The following reagents were used: Doxorubicin hydrochloride (Sigma-Aldrich, Tokyo, Japan), triethylamine (Et_3_N), and 2-(1H-benzotriazol-1-yl)-1,1,3,3-tetramethyluronium hexafluorophosphate (HBTU) (Sigma-Aldrich, Saint Louis, MO, USA). Solvents: dichloromethane, methanol, and dimethylformamide (DMF) (Khimmed, RF, Moscow, Russia). The solvents were purified and prepared using standard techniques.

L-Histidine, 4-nitroso-N,N-dimethylaniline (RNO), Cremophor EL (CrEL), sodium azide, and Hoechst 33342 were supplied by Sigma Chemical Co. (St. Louis, MO, USA). Rose Bengal (RB) was purchased from DiaM (Moscow, Russia). LysoTracker Green (LG) was supplied by Molecular Probes (Eugene, OR, USA).

The following equipment was used: Sartorius electronic analytical balance with a weighing accuracy of 0.0001 g (Germany); IKA rotary evaporator (Germany); a CM-6M.06 medical centrifuge (Latvia); Minispin microcentrifuge (Eppendorf, Framingham, MA, USA); Ultrospec 2100 Pro spectrophotometer (GE Healthcare, Chicago, IL, USA); Elmasonic S100H ultrasonic bath (Elma, Singen, Germany); Dionex UltiMate RS 3000 liquid chromatographic system coupled with a Q-Exactive high-resolution hybrid mass spectrometer (Thermo Scientific, Waltham, MA, USA); LESA-01-Biospec unit for local spectroscopy and photodynamic diagnostics (BIOSPEK, Moscow, Russia); Latus semiconductor laser device (CJSC Semiconductor Devices, St. Petersburg, Russia); ALUGRAM Xtra SIL G/UV254 plates for analytical TLC coated with silica gel 60 (0.2 mm) (Macherey-Nagel, Düren, Germany). NMR spectra were recorded with a Bruker DPX300 spectrometer in CDCl_3_. The residual signals of ^1^H nuclei were used for scale calibration. The experiments were performed according to the standard Bruker procedures.

### 3.1. Synthesis of The Bacteriochlorin Conjugate with Doxorubicin

PS (Scheme 1), which is a derivative of natural bacteriochlorin, was prepared according to the technique that we reported previously [36].

To synthesize PS+DOX, a solution of doxorubicin hydrochloride (1 equiv., 0.0301 mmol, 18.0 mg) and triethylamine (8.4 equiv., 0.2518 mmol, 35 μL) in DMF was added dropwise to a solution of PS (1 equiv., 0.0301 mmol, 20.0 mg) and 2-(1H-benzotriazol-1-yl)-1,1,3,3,3-tetramethyluronium hexafluorophosphate (HBTU) (6.7 equiv., 0.2010 mmol, 76.2 mg) in DMF (5 mL). The reaction was carried out in argon atmosphere in the dark at room temperature for 12 h. After removal of the solvent under reduced pressure in a rotary evaporator, the product was purified by preparative column chromatography on silica gel in the dichloromethane–methanol system with a methanol concentration gradient from 30 to 60 vol.%. The yield of PS+DOX (Scheme 1) was 50.0% (18.4 mg).

¹H NMR spectrum (300 MHz, CDCl_3_, δ) of PS+DOX:

Bacteriochlorin fragment:

8.56 (H, s, 10-H), 8.51 (H, s, 5-H), 8.32 (H, s, 20-H), 5.05 (H, m, 17-H), 4.43 (4H, m, 3^4^-CH_2_, 13^3^-CH_2_), 4.19 (3H, m, 7-H, 18-H), 3.08 (H, m, 8-H), 3.58 (3H, s, 12-CH_3_), 3.23 (3H, s, 2-CH_3_), 2.71 (3H, s, 3^1^-CH_3_), 2.38 (4H, m, 17^2a^-CH_2_, 17^1a^-CH_2_, 17^2b^-CH_2_, 17^1b^-CH_2_), 2.03 (6H, m, 3^5^-CH_2_, 13^4^-CH_2_, 8^1^-CH_2_), 1.79 (3H, d, *J* = 7.2 Hz, 7-CH_3_), 1.64 (3H, d, *J* = 7.1 Hz, 18^1^-CH_3_), 1.18–1.05 (9H, m, 13^5^-CH_3_, 3^6^-CH_3_, 8^2^-CH_3_), 0.20 (s, NH), −0.20 (s, NH).

Doxorubicin fragment:

13.95 (H, s, 11-OH), 13.24 (H, s, 6-OH), 8.11 (H, d, *J* = 7.4 Hz, 1-CH), 7.96 (H, d, *J* = 7.4 Hz, 2-CH), 7.71 (H, t, *J* = 6.8 Hz, -NH), 7.54 (H, d, *J* = 6.4 Hz, 3-CH), 5.57 (H, s, 9-OH), 5.33 (1H, m, 1′-CH), 4.92 (H, m, 7-CH), 4.73 (2H, s, 14-OH, 4′-OH), 4.70 (2H, m, 14-CH_2_), 4.09 (H, m, 5’-CH), 4.01 (3H, s, -OCH_3_), 3.72 (H, m, 3’-CH), 3.32 (H, s, 4’-CH), 3.08 (2H, d, *J* = 7.1 Hz, 10-CH_2_), 2.12 (2H, m, 8-CH_2_), 1.82 and 1.49 (2H, m, 2’-CH_2_), 0.89 (3H, m, 6’-CH_3_).

Mass spectrum: *m*/*z* value calculated for C_66_H_77_N_7_O_18_ [M+H]^+^—1223.54; found—1223.5375 [M+H]^+^.

### 3.2. Detection of The Quantum Yield of Singlet Oxygen Generation

The measurements and calculations of φ were performed as described previously [43]. Air-equilibrated samples of PS+DOX (18 μM in 1% CrEL) and RB (4 μM in 1% CrEL) were supplemented with RNO (30 μM) and histidine (11 mM) in a sodium phosphate buffer (pH 7.4) and irradiated with a laser (532 nm, 0.82 mW, 40 min). Absorption spectra were measured every 10 min. The concentrations of the standard RB solution and solution of PS+DOX were adjusted to provide equal absorption of RB and the bacteriochlorin moiety of PS+DOX (0.09 optical unit cm^−1^) at 532 nm.

To confirm singlet oxygen generation, sodium azide (20 mM) was added to the reaction mixture in a control experiment.

The percentage α of bleached RNO molecules was calculated using the formula:

(1) $$\alpha =\frac{A_{0}-A}{A_{0}}\cdot 100\%$$

where *A*_0_ and *A* are absorptions of RNO at 440 nm initially and during irradiation, respectively.

Based on the measurement results, a linear graph of the dependence of α on the irradiation time was constructed. The quantum yield of singlet oxygen generation (φ) was calculated using the formula:

φ (^1^O_2_) = φRB · tgα(t)/tgαRB(t) · (1–10^−(ARB·L)^)/(1–10^−(A·L)^) (2)

where tgα(t) and tgαRB(t) are the tangents of the slope angles of the dependences α on the irradiation time for PS+DOX and RB, respectively; A and ARB are the optical densities at 532 nm of the solutions with PS+DOX and RB, respectively; L is the length of the optical path of the cuvette (1 cm).

### 3.3. Chromatography–Mass Spectrometry

Chromatographic analysis of PS+DOX was performed with a Dionex UltiMate RS 3000 liquid chromatographic system coupled with a high-resolution Q-Exactive hybrid mass spectrometer (Thermo Scientific, USA).

Sample components were separated using a “Pyramid” reversed-phase column with C-18 grafted phase. Column length 75 mm, inner diameter 2 mm, sorbent particle diameter 3 microns, manufactured by Macherey-Nagel, Germany (cat. no. 37669012).

Mili Q deionized water (18.2 cm^−1^) was used as component A of the mobile phase (MP). Isopropyl alcohol of HPLC grade from Carlo Erba, France (cat. no. UN 1219), was used as component B of the mobile phase. Methyl alcohol for HPLC from Fisher Chemical, Taiwan (cat. no. 1923759), was used as the washing solution for the autosampler. The MP flow rate was 0.400 mL. The gradient elution mode was used. The relative content of MP B (%) was varied as follows during the analysis: 0.00–1.00 min (5%); 1.00–12.00 min (95%); 12.00–18.00 min (95%); 18.00–18.01 min (5%); 18.01–20.00 min (5%). The temperature of the column thermostat was 40 °C. The analysis time was 20.00 min. The volume of the aliquot injected in the column was 3.00 µL.

Compounds were detected in the positive ion mode using heated electrospray ionization (HESI). The atomizing gas (N_2_) flow rate was 45 arb. units, the auxiliary gas flow rate was 25 arb. units, and the drying gas flow rate was 5 arb. units. The spray capillary voltage was 4.0 kV. The atomizing capillary temperature was 200 °C. The temperature of the input capillary was 350 °C. The voltage on the S lens was 50 arb. units. The detection range of *m*/*z* values was 350–2200 arb. units. The resolution was 60.000 arb. units. Mass spectra were recorded in “Profile” mode.

### 3.4. In Silico Studies of The Resulting PS+DOX

Molecular docking studies for DNA were performed using HEX 8.0.0 software (Dave Ritchie, Paris, France). Receptor preparation was performed in MGL tools (The Scripps Research Institute, San Diego, CA, USA). The parameters used for docking were as follows: Shape+Electro correlation type, OPLS Minimisation post-processing, FFT 3D mode, grid size 0.6, receptors range 180, ligands range 180, rotation range 360, and distance range 40. Molecular docking studies for protein were performed using Autodock Vina [37]. The parameters used for docking were as follows: exhaustiveness = 16. Visualization and determination of interactions were performed in BIOVIA Discovery Studio Visualizer (Dassault Systèmes SE, Paris, France).

### 3.5. In Vitro Study 

#### 3.5.1. Characteristics of in Vitro Test Systems

The following cell lines were used to assess photoinduced and dark activity:

- MCF-7–human breast adenocarcinoma (collection of the Institute of Cytology of the Russian Academy of Sciences);
- 4T1–mouse mammary gland carcinoma (ATCC collection).

MCF-7 cells were cultured in Eagle MEM medium (PanEco, Russia), and 4T1 cells in RPMI1640 medium (PanEco, Moscow, Russia) supplemented with PS+DOX mM-L-glutamine (PanEco, Moscow, Russia) and 10% fetal bovine serum (FBS) (Biowest, South America); in 25 cm^2^ culture flasks (SPL Life Sciences, Pochon, Republic of Korea) at 37 °C in a humid atmosphere with 5% carbon dioxide. During passaging, Versene solution (PanEco, Moscow, Russia) was used to remove cells from the substrate. For in vitro studies, cells of the 3rd to 5th passages with an optimal seed concentration of 6 × 10^4^ cells/mL were used.

#### 3.5.2. Cellular Imaging of the PS+DOX Studied In Vitro

Cells were subcultured two times per week. For microscopic experiments, cells were seeded (2 × 10^5^ cells per ml, per well) on round cover glasses placed in 24-well plates and grown for 24 h.

The intracellular distribution of PS+DOX in cells was studied using the LSM-710 confocal laser scanning microscope (Carl Zeiss AG, Oberkochen, Germany) with APD detection. The confocal fluorescent images were obtained with an α-Plan-Apochromat 100×/1.4 oil-immersion objective at 0.3 µm lateral and 1.5 µm axial resolution.

To study intracellular distribution, 3–10 μM of PS+DOX was added to MCF-7 or 4T1 cells from a 0.5 mM stock solution in 1% emulsion of CrEL. Cells were incubated with PS+DOX for 4 or 24 h. Fluorescence intensity of PS+DOX in cells was excited at the 543 nm wavelength and recorded in the spectral range provided by an LP655 filter (it transmits a signal at lengths of more than 655 nm). Fluorescence intensity of Hoechst 33342 in cells was excited at 405 nm wavelength and recorded in the 470–520 nm spectral range using an FEO-detection system.

To check the accumulation of PS+DOX in lysosomes, cells were incubated with 50 nM LG (30 min, 37 °C) after incubation with PS+DOX (5 μM for 4 h, 37 °C) and studied using CLSM (APD-detection system). Fluorescence of LG was excited at 488 nm and imaged within the 505–530 nm range. Fluorescence of PS+DOX was excited at 543 nm and imaged at lengths above 655 nm. Control cells were separately incubated with PS+DOX or LG and measured under the same conditions. The spectral crosstalk coefficient was 0.13 for LG in the >655 nm range. For PS+DOX, the spectral crosstalk coefficient was <0.01 in the 505–530 nm range. Fluorescent CLSM images of intracellular distribution of PS+DOX and LG were corrected for the spectral crosstalk.

#### 3.5.3. Methodology for Studying Photoinduced and Cytotoxic Activity in an In Vitro System

To study photoinduced and cytotoxic activity, tumor cells were seeded in 96-well plates in an amount of 6 × 10^3^ cells per well in 100 μL of complete growth medium. Cell exposure was performed in the exponential phase of cell growth, 24 h after seeding. Solutions of compounds were placed in wells at final concentrations ranging from 24.5 to 0.040 μM (PS and PS+DOX) and 1233.0 to 2.7 μM (DOX). To estimate photoinduced activity, cells were incubated with the compounds for 4 h and irradiated with a halogen lamp through a broadband filter (NIOPIK, Moscow, Russia) at a light dose of 10 J/cm^2^. The photoinduced activity of PS was estimated 24 h after irradiation.

To estimate the combined effect, cells with the conjugate were incubated for 4 h and irradiated under the same conditions, then incubated for 72 h under standard conditions, and the combined activity was estimated.

To estimate the cytotoxic effect, cells with PS, PS+DOX, and DOX were added into wells at the same concentrations and incubated for 72 h without irradiation.

Culture medium containing 10% fetal calf serum was used as the control.

Cell survival was estimated using the colorimetric MTT test. Inhibition of cell growth in the culture by more than 50% (IC50) was considered a biologically significant effect. Quantitative parameters were calculated from three independent tests.

### 3.6. In Vivo Study

#### 3.6.1. Animals

To evaluate the antitumor efficacy, we performed a pilot PDT study with sarcoma M-1-bearing Wistar rats three months of age weighing 180–200 g on average. The animals were purchased from the Biomedical Technology Scientific Center of the Federal Biomedical Agency of Russia (Moscow) and housed in T-4 cages under natural light conditions with forced ventilation of 16 times·h^−1^, at room temperature and at 40–70% relative humidity. The rats had free access to water and PK-120–1 food for rodents (Laboratorsnab Ltd., Moscow, Russia).

All the experiments in animals were carried out according to the guidelines for the care and use of laboratory animals of the National Medical Research Radiological Center of the Ministry of Health of the Russian Federation and in accordance with the rules and requirements of the European Convention ETS/STE N 123 and the GLP international standard (OECD Guide 1:1998). The animal experimental protocols were approved by the Ethical Committee for Animal Experiments of the National Medical Research Radiological Center (N 1-SI-00051) [43,44].

#### 3.6.2. Tumor Model

The antitumor activity was studied using the rat sarcoma M-1 model. The tumor strain was obtained from the tumor bank of the N.N. Blokhin National Medical Research Center of Oncology of the Ministry of Health of the Russian Federation.

For the experiments, 30 mg of crushed tumor tissue was transplanted on the outer side of the thigh. The experiment was started 8–9 days after transplantation when the largest diameter of a tumor node reached 0.8–1.0 cm.

#### 3.6.3. Biodistribution

The accumulation of the drug in the tumor and in the surrounding healthy tissues was studied by laser spectrometry using an LESA-01-Biospec instrument, Russia. The concentrations of the drugs were estimated by fluorescence intensity. PS, PS+DOX, and DOX were infused intravenously at a dose of 5.0 mg/kg. Selectivity was determined by the contrast index (tumor/healthy tissue). The first measurement was performed before drug administration (0 min), then every 30 min up to 180 min. Determination of the drug concentrations and the dynamics of their amounts in tissues in vivo is necessary for determining the optimal time from the moment the photosensitizer is administered until the laser treatment. Every experimental group comprised 4 animals.

#### 3.6.4. Study of PDT Efficiency

The antitumor efficacy of PDT with PS, PS+DOX, and DOX was studied using M-1 rat tumors. Every experimental group consisted of 6 animals (Table 4).

A Latus semiconductor laser device manufactured by CJSC “Semiconductor Devices” (St. Petersburg) with an emission wavelength of 810 nm was used as the laser radiation source. The time interval before irradiation was determined from biodistribution and fluorescence contrast results and ranged from 90 to 120 min. Irradiation modes: power density Ps = 0.48 W/cm^2^, energy density E = 150 J/cm^2^. The tumor of an animal was irradiated by a single light beam with a diameter of 1.5–1.8 cm that completely covered the tumor and the surrounding tissue for a width of 2–4 mm. All the rats were anesthetized with a combination of zoletil 100 (Virbac, Carros, France) and xylazine 2% (Alfasan International B.V., Venray, The Netherlands) administered intraperitoneally 10 or 15 min before PDT.

#### 3.6.5. Study of Chemotherapy Efficiency

The DOX chemotherapeutic agent was administered intravenously to rats once at a dose of 2.24 mg/kg into the tail vein. The experimental group comprised 6 animals (Table 4).

#### 3.6.6. Estimation of Antitumor Efficacy

The presence of tumor nodules and their volumes were determined during the observation. Tumor measurements began 4 or 5 days after PDT when the edema decreased. The tumor volume was estimated as V = d_1_ × d_2_ × d_3_ × 0.52, where d_1_, d_2_, and d_3_ were the three orthogonal diameters of the tumor nodule. The tumor size was recorded for 28 days.

The observation was continued for 90 days after the treatment, then the animals were euthanized, and the presence or absence of the tumor mass was assessed macroscopically. The absence of a tumor node in 90 days after the treatment was considered remission.

The efficacy factors for this study included the following:

Tumor growth inhibition (TGI), TGI = [(Vc − Ve)/Vc] × 100%, where Ve and Vc are mean tumor volumes in the treated tumor-bearing mice and control tumor-bearing mice, respectively. TGI was calculated 24 and 28 days after treatment;

The number of remissions (RN), RN = [Nc/Nt] × 100%, where Nc is the total number of cured animals and Nt is the total number of treated animals. A drug was considered highly efficient at RN ≥ 50%;

The mean animal life expectancy (MLE, days) and the life expectancy increase (LEI%) compared to the control were calculated. The LEI is considered significant if LEI ≥ 50% [45].

(3) $$LEI=\frac{MLEtest-MLEcontrol}{MLEcontrol}\cdot 100\%.$$

#### 3.6.7. Statistics

Quantitative data were expressed as the mean ± confidence interval. In the in vitro experiments, statistical analysis was performed with GraphPad Prism 8.0 (GraphPad Software Inc., San Diego, CA, USA) using one-way ANOVA analysis of variance. The results were confirmed by Bartlett’s test. In the in vivo experiments, Mann–Whitney U criteria were used to estimate the differences in the quantitative parameters between the groups. The calculations were performed using Statistica 8.0 (StatSoft, Inc., Tulsa, OK, USA). The difference was considered significant at *p* < 0.05.

## 4. Conclusions

In this work, a hitherto unknown conjugate of doxorubicin and natural bacteriochlorin with absorption in the region of 800 nm was obtained. The purity and structure of the compound obtained were reliably confirmed by a set of physicochemical methods of analysis, which allowed this compound to be used for the estimation of in vitro and in vivo antitumor activity. We also conducted in silico studies of the interaction of the resulting compound and a DNA fragment.

In vitro studies have shown that the conjugate of bacteriochlorin and Doxorubicin (PS+DOX) has pronounced antitumor activity that is greater than that of Doxorubicin itself. This effect can be explained by the combined effect of photodynamic and chemotherapy.

Similarly to bacteriochlorins [46], the intracellular distribution of PS+DOX occurs in both types of cells; similar intracellular distributions of the compound are observed: accumulation in vesicular structures, intense diffuse staining of the cytoplasm, and much weaker staining of the nuclei.

It was found that the most efficient method for the treatment of M-1 sarcoma tissue implanted in rats was to use the conjugate of PS+DOX (treatment schedule III). A pronounced positive effect was obtained, namely, complete eradication of tumor nodes in 83.3% of animals on the 90th day after the treatment and a significant increase in the life expectancy in rats compared to the control group. Treatment schedule IV, i.e., combined therapy (PDT with PS and chemotherapy with DOX) using the same doses and the same parameters of laser exposure, was also highly efficient. Complete regression of the tumor on the 90th day after the therapy was observed in 50.0% of the animals.

The photoinduced generation of singlet oxygen for PS+DOX was 0.45 ± 0.03, which indicates the absence of a negative effect of the doxorubicin fragment on the photophysical properties of the conjugate (the φ values for bacteriochlorophyll derivatives are 0.5–0.6) [47].

Thus, PS+DOX can be considered as a potential drug for the implementation of combined photodynamic and chemotherapy in oncology.

## Figures and Tables

**Table 1:** Estimated interaction energies of ligands and DNA.

Compound’s E, kJ/mol	E_total_	E_shape_	H-Bonds
DOX	−303.00	−319.80	6
PS+DOX	−305.90	−333.40	8 *

* Note: the maximum number of hydrogen bonds.

**Table 2:** Photoinduced and cytotoxic activity of PS, PS+DOX, and DOX in vitro.

IC50, nM			
Compound	Photoinduced Activity	Combined Activity	Cytotoxic Activity
	4 h PS Incubation with Cells → Irradiation at 10 J/cm^2^ → MTT after 24 h	4 h PS Incubation with Cells → Irradiation at 10 J/cm^2^ → MTT after 72 h	72 h Incubation of Compounds with Cells → MTT
MCF-7—Human Breast Adenocarcinoma			
PS	707 ± 16 nM	691 ± 14 nM	10643 ± 68 nM
PS+DOX	671 ± 14 nM	442 ± 11 nM	8672 ± 49 nM
DOX	–	–	6237 ± 96 nM
4T1—Mouse Breast Carcinoma			
PS	194 ± 10 nM	188 ± 9 nM	8056 ± 51 nM
PS+DOX	188 ± 8 nM	123 ± 7 nM	295 ± 15 nM
DOX	–	–	5525 ± 71 nM

**Table 3:** Antitumor efficiency of different therapy schedules for M-1 sarcomas in rats.

Group No.	Agent Dose, mg/kg	MLE, Days	LEI, %	RN, %
Schedule I: monophotodynamic therapy with PS				
1	PS 2.76	50.2 ± 15.8	54.4	16.7
Schedule II: monochemotherapy with DOX				
2	DOX 2.24	53.5 ± 22.6	64.6	33.2
Schedule III: combined therapy with PS+DOX				
3	PS+DOX 5.00 (2.76 (PS) + 2.24 (DOX))	81.3 ± 17.0	150.3	83.3
Schedule IV: PDT + chemotherapy with administration of PS and DOX				
4	5.00 (2.76 (PS) and 2.24 (DOX))	65.2 ± 21.9	100.5	50.0
5	Control	32.5 ± 1.8	–	0

Note: the mean animal life expectancy (MLE, days), the life expectancy increase (LEI, %); LEI ≥ 50%—a significant increase in the life expectancy of rats with tumor compared to the control; the number of remissions (RN), RN = [Nc/Nt] × 100%; E = 150 J/cm^2^; Ps = 0.48 W/cm^2^.

**Table 4:** Study design.

Group No.	Agent	Dose, mg/kg	Administration Method	Irradiation Parameters	
				E, J/cm^2^	Ps, W/cm^2^
Treatment Schedule I: Monophotodynamic Therapy with PS, Latus Laser, λ = 810 nm					
1	PS	2.76	Intravenously	150	0.48
Treatment schedule II: monochemotherapy with DOX					
2	DOX	2.24	Intravenously	–	–
Treatment schedule III: combined therapy with PS+DOX, Latus laser, λ = 810 nm					
3	PS+DOX	5.0 (2.76 (PS) + 2.24 (DOX))	Intravenously	150	0.48
Treatment schedule IV: PDT + chemotherapy with administration of PS and DOX, Latus laser, λ = 810 nm					
4	PS and DOX	5.0 (2.76 (PS) and 2.24 (DOX))	Intravenously	150	0.48
5 Control	0.9% NaCl	5.0	Intravenously	–	

## Data Availability

The data presented in this study are available from the authors.
